# New insights on the ventral attention network: Active suppression and involuntary recruitment during a bimodal task

**DOI:** 10.1002/hbm.25322

**Published:** 2020-12-21

**Authors:** Rodolfo Solís‐Vivanco, Ole Jensen, Mathilde Bonnefond

**Affiliations:** ^1^ Laboratory of Neuropsychology Instituto Nacional de Neurología y Neurocirugía Manuel Velasco Suárez Mexico City Mexico; ^2^ Donders Institute for Brain, Cognition and Behaviour Centre for Cognitive Neuroimaging, Radboud University Nijmegen Netherlands; ^3^ Centre for Human Brain Health University of Birmingham Birmingham United Kingdom; ^4^ Computation, Cognition and Neurophysiology team (Cophy), INSERM U1028, CNRS UMR5292 Lyon Neuroscience Research Center (CRNL) Bron Cedex France

**Keywords:** alpha oscillations, attention, dorsal attention network, multisensory integration, ventral attention network

## Abstract

Detection of unexpected, yet relevant events is essential in daily life. fMRI studies have revealed the involvement of the ventral attention network (VAN), including the temporo‐parietal junction (TPJ), in such process. In this MEG study with 34 participants (17 women), we used a bimodal (visual/auditory) attention task to determine the neuronal dynamics associated with suppression of the activity of the VAN during top‐down attention and its recruitment when information from the unattended sensory modality is involuntarily integrated. We observed an anticipatory power increase of alpha/beta oscillations (12–20 Hz, previously associated with functional inhibition) in the VAN following a cue indicating the modality to attend. Stronger VAN power increases were associated with better task performance, suggesting that the VAN suppression prevents shifting attention to distractors. Moreover, the TPJ was synchronized with the frontal eye field in that frequency band, indicating that the dorsal attention network (DAN) might participate in such suppression. Furthermore, we found a 12–20 Hz power decrease and enhanced synchronization, in both the VAN and DAN, when information between sensory modalities was congruent, suggesting an involvement of these networks when attention is involuntarily enhanced due to multisensory integration. Our results show that effective multimodal attentional allocation includes the modulation of the VAN and DAN through upper‐alpha/beta oscillations. Altogether these results indicate that the suppressing role of alpha/beta oscillations might operate beyond sensory regions.

## Graphical abstract

Detection of unexpected, yet relevant events from multiple sensory sources is essential in daily life. We explored the dynamics of the ventral attention network (VAN), a set of brain regions related to the processing of unattended but salient stimuli, when relevant information was anticipated (i.e., during top‐down attention) and when unexpected congruent information from another sensory modality was presented (involuntary attentional enhancement). We report that activity in the alpha/beta range (12–20 Hz) within the VAN indexed both top‐down and attentional enhancement processes. In addition, the VAN was synchronized with the dorsal attention network in this frequency range, suggesting an integrated role of both networks for attentional regulation. Our results shed light on the neurophysiological mechanisms that the brain carry out for orienting attention to relevant environmental stimuli.

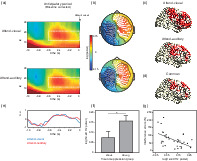

## 1 INTRODUCTION

The capacity of allocating attention toward specific stimuli, even involuntarily, is crucial for selecting relevant information and ensuring optimal behavior in daily life. The dorsal and ventral attention networks (DAN and VAN) have been shown to be involved in such processes as revealed by functional magnetic resonance imaging (fMRI). The DAN comprising the frontal eye fields (FEF), the superior parietal lobules (SPL), and the inferior parietal sulci (IPS), is involved in top‐down attentional allocation while the VAN, encompassing the right temporoparietal junction (TPJ) and ventral frontal cortex (VFC), allows orienting attention toward unattended relevant or salient stimuli (see Corbetta, Patel, & Shulman, 2008 for reviews; Corbetta & Shulman, 2002; Vossel, Geng, & Fink, 2014).

The oscillatory dynamic of the DAN and sensory areas during top‐down attention tasks have also been studied using electroencephalography (EEG), magnetoencephalography (MEG), and transcranial stimulation (e.g., Banerjee, Snyder, Molholm, & Foxe, 2011; Doesburg, Bedo, & Ward, 2016; Horschig, Jensen, van Schouwenburg, Cools, & Bonnefond, 2014; Marshall, O'Shea, Jensen, & Bergmann, 2015; Popov, Kastner, & Jensen, 2017; Rohenkohl & Nobre, 2011; Sadaghiani et al., 2012; Sauseng, Feldheim, Freunberger, & Hummel, 2011; Siegel, Donner, Oostenveld, Fries, & Engel, 2008; Worden, Foxe, Wang, & Simpson, 2000) while, to the best of our knowledge, very few electrophysiological studies focused on the oscillatory dynamic of the VAN during involuntary allocation of attention (ElShafei, Fornoni, Masson, Bertrand, & Bidet‐Caulet, 2018; Proskovec, Heinrichs‐Graham, Wiesman, McDermott, & Wilson, 2018; Sauseng et al., 2005). These studies have reported theta (4–8 Hz) and alpha/beta (8–20 Hz) decreases in the DAN and VAN during orienting of attention to relevant stimuli, or a gamma (> 40 Hz) increase in the VAN during the presentation of distracting (irrelevant) sounds to be ignored. However, the dynamic of the inhibition of the VAN as reported in some fMRI studies during top‐down attention has not been studied. In addition, whether the VAN is activated when involuntary attentional enhancement results from multisensory integration remains unknown. The goal of the present study is (a) to characterize the oscillatory dynamics associated with the suppression of the activity of the VAN during top‐down attention and (b) to reveal for the first time the potential additional role of this network in involuntary enhancement of attention across sensory modalities.

fMRI studies have revealed that during top‐down attentional processes or during short‐term memory involving high memory load, the activity in the TPJ is suppressed (Shulman et al., 2003; Shulman, Astafiev, McAvoy, d'Avossa, & Corbetta, 2007; Todd, Fougnie, & Marois, 2005), suggesting that inactivation of TPJ activity protects goal‐driven behavior from distractors. Based on the literature indicating a role of alpha oscillations in functional inhibition (Bonnefond & Jensen, 2012, 2013, 2015; Bonnefond, Kastner, & Jensen, 2017; Foxe & Snyder, 2011; Jensen & Mazaheri, 2010; Klimesch, Sauseng, & Hanslmayr, 2007), we predict that alpha oscillations will be high in the TPJ following the presentation of a cue directing attention to a specific sensory modality.

In addition, some studies have focused on the involvement of the VAN in supramodal attention tasks (Macaluso, 2010; Macaluso, Frith, & Driver, 2002), as it can be involuntarily activated by irrelevant stimuli coming from sensory modalities not to be attended (e.g., auditory) but spatially congruent to relevant information (e.g., visual) (Santangelo, Olivetti Belardinelli, Spence, & Macaluso, 2009). In the present study, we wanted to determine whether the VAN is also recruited when information coming from an unattended sensory modality (e.g., visual) is congruent with the attended one (e.g., auditory). This process is different from the traditional reorientation of attention as studied in Posner tasks, as it does not involve a full switch of attention from one location (or modality) to another. It is expected rather to involve an involuntary attentional enhancement for the target due to its increased saliency, triggered by congruency between sensory domains (Gau, Bazin, Trampel, Turner, & Noppeney, 2020). Such enhancement should be expressed behaviorally as an improved performance given by congruency across modalities, instead of a cost given by attentional switch from one to the other. With a bimodal attention task, we hypothesized that the VAN would be recruited in the congruent trials of both attention conditions (visual or auditory), as reflected by a decrease of alpha oscillations in the TPJ (indicating a release from suppression, see e.g., Solis‐Vivanco, Rodriguez‐Violante, Cervantes‐Arriaga, Justo‐Guillen, and Ricardo‐Garcell (2018)) and/or an increase of gamma or theta oscillations (ElShafei et al., 2018; Proskovec et al., 2018; Sauseng et al., 2005). Such recruitment in congruent trials was further expected to be related to task performance.

## 2 MATERIALS AND METHODS

### 2.1 Subjects

We included 36 healthy subjects attending college who were recruited from Radboud University's research participation scheme. Inclusion criteria for all participants included Dutch as their mother tongue, right‐handedness according to the Edinburgh Handedness Inventory (Oldfield, 1971), normal or corrected‐to‐normal vision, and reported normal audition. Participants with a psychiatric or neurological diagnosis were excluded. Two participants were excluded due to excessive noise or movement artifacts during MEG recordings. The final sample consisted of 17 females and 17 males, with a mean age of 23 ± 2.5 years. The study was conducted at the Donders Institute for Brain, Cognition and Behaviour and fulfilled the Declaration of Helsinki criteria (WMA, 2013).

### 2.2 Experimental design

**FIGURE 1:**
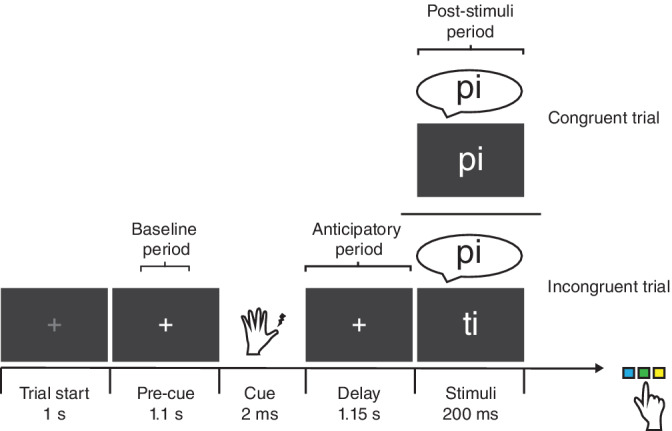
Experimental paradigm. After a lateralized somatosensory cue indicating which sensory domain to attend (visual or auditory), participants were asked to press one of three buttons according to the relevant stimulus in that domain. Baseline, anticipatory, and poststimuli (congruency) time periods are indicated

A cross‐modal attention task was designed using MATLAB (MathWorks) custom scripts and Psychtoolbox (psychtoolbox.org). Each trial (~5 s duration) began with a black background and a gray central fixation cross that lasted for 1 s and was projected on an acrylic screen by an EIKI LC‐XL100L projector with a resolution of 1024 × 768 and a refresh rate of 60 Hz that lasted for 1 s (Figure 1). Subjects were asked to blink or move their eyes only during this period. Afterwards, the fixation cross turned white and 1,100 ms later an electro‐tactile cue (2 ms) was delivered to the left or right thumb. This cue instructed the participants to allocate attention to the visual (Attend‐visual condition; 50% of trials) or auditory (Attend‐auditory condition; 50% of trials) stimuli, respectively. The cue was administered with two constant current high voltage stimulators (type DS7A, Digitimer, Hertfordshire, UK; mean current = 3.83 mA). After a post‐cue interval of 1,150 ms, visual and auditory stimuli were presented simultaneously for 200 ms, and they consisted of three syllables without meaning in Dutch. They were formed by a plosive consonant and the same vowel (“pi,” “ti,” and “ki”). The timing of the stimuli onset and their duration was carefully controlled. The use of the same vowel (“i”) in all stimuli further allowed us to guarantee that the length of the syllables was stable.

Each syllable was delivered with the same probability in both sensory domains. From the total number of trials (798), roughly 75% (599) were incongruent (different syllable between visual and auditory modality) and 25% (199) were congruent (same syllable in both modalities). The higher number of incongruent trials was originally planned to promote anticipatory suppression of distracting information from the irrelevant sensory modality, as this was the main objective of a previous report in which we showed that alpha oscillations can be phase adjusted in anticipation of relevant stimuli at sensory regions (Solis‐Vivanco, Jensen, & Bonnefond, 2018). Nevertheless, congruent trials were included in order to explore VAN recruitment, therefore, the focus for this study was beyond sensory areas. Moreover, this proportion resembles oddball tasks used to explore TPJ activation by infrequent stimuli (Corbetta & Shulman, 2002). Visual stimuli were presented at the center of the screen in white. Auditory stimuli were digitally created using a male voice and delivered from the computer controlling the task to plastic ear‐tubes adapted to MEG recordings and inserted in participants' ear canals. Each syllable was associated with either one of three buttons in a response pad. Participants were asked to respond as accurate and fast as possible to the syllable in the modality they were instructed to attend in each trial, by pressing the corresponding button using their right index, middle, or ring finger. The correspondence between the side of the cue and the modality to attend, and the assigned syllables to the buttons were counterbalanced across participants. All trials were randomly distributed across participants. Five breaks were introduced in the experiment, in which participants were informed about their performance. Reaction times (RT) and response accuracy were recorded along the experiment. Participants were given up to 1,500 ms to respond. During both the training and experimental sessions, clear perception of stimuli was verified in each participant. In addition, although the MEG system we used provides appropriate prescription glasses to be used during acquisition for vision correction, none of our participants needed them.

### 2.3 Data acquisition

We used a whole‐head magnetoencephalography (MEG) system with 275 axial gradiometers (VSM/CTF systems, Port Coquitlam, Canada) housed in a magnetically shielded room. MEG recordings were sampled at 1200 Hz with an online 300 Hz low‐pass filter. The signal was down‐sampled to 600 Hz afterwards for off‐line analysis. No additional bandpass filtering was applied for any analysis, in order to preserve the possibility to perform analyses in the whole spectrum. All participants were recorded in the supine position. Coils placed at the nasion and the left and right ear canals were used to measure participants' head location relative to the MEG sensors during the experiment. During the recordings, an Eyelink 1,000 eye tracker (SR Research, Ontario, Canada) was used to monitor eye movements and blinks. Additionally, we used a FASTRAK device (Polhemus, VT) to record the head shape of participants with 300 head points relative to the three fiducial points (nasion and the left and right ear canals). In addition to the MEG recordings, a structural magnetic resonance image (MRI) of the participants' brain was acquired using a 3 T Siemens Trio system (Erlangen, Germany) and with a voxel size of 1 mm^3^. During the MRI acquisition, earplugs with a drop of Vitamin E in place of the coils were used for co‐registration of the MRI and MEG data.

### 2.4 Procedure

The experiment was conducted over three sessions for all participants. During the first session, inclusion criteria were confirmed, general information about the study and informed consent letters were provided, and detailed instructions about the experiment were presented. Participants then performed a practice session with 150 trials inside the MEG room. During the second session, the participants' head shape was digitized, and the actual MEG experiment was conducted. During the third session, the MRI was obtained. All data are available by request to the authors.

### 2.5 Data analysis

All data analyses were done using MATLAB custom scripts and the Fieldtrip toolbox (Oostenveld, Fries, Maris, & Schoffelen, 2011). Epochs of the MEG recording extending 2 s before and 1 s after the onset of visual and auditory stimuli were extracted. Only epochs containing correct responses were considered for further signal analyses. Special care was taken to identify and remove artifact activity. Trials containing muscle artifacts, superconducting quantum interference device (SQUID) jumps or eye blinks and saccades (as shown by the eye tracker signal), were rejected using an automatic routine based on mean z‐scores across sensors exceeding a threshold given by the data variance within each participant (cut‐off of ± 2 *SEM*). Since visual stimuli were always at the center of the screen and no visual search was needed, long eye movements were highly infrequent. We trained our participants to blink exclusively after giving their response in each trial, so artifacts due to blinking were particularly infrequent as well. Additional visual inspection was applied to the remaining trials before demeaning and including them in further analyses. The mean number of trials included in the analysis was 558 ± 112, with no significant differences between Attend‐visual and Attend‐auditory conditions (282 ± 61 vs. 277 ± 62, F_[1,32]_ = 0.07, *p* = .80), nor an interaction of condition by congruency (Incongruent: 183 ± 46 vs. 183 ± 49, Congruent: 65 ± 16 vs. 67 ± 15, F_[1,32]_ = 0.17, *p* = .69). Although the difference in number of trials between congruent and incongruent stimuli might produce unbalanced signal‐to‐noise ratios, it should be noted that reducing the number of trials from one condition to resemble the number of the other one reduces statistical power and increases the Type II error rate (likelihood of accepting the null hypothesis when it is false). Moreover, unbalanced number of trials does not increase the Type I error rate (likelihood of rejecting the null hypothesis when it is true), especially when using mean values (e.g., across time and frequencies), which was our case (Luck, 2014).

Epochs were analyzed at sensor and source level. The analyses performed at sensor level are essential to identify target time and frequency ranges. Source analyses were then used to determine the source origin of the effects observed at sensor level and to explore the oscillatory activity in specific regions of interest. For the sensor‐level analyses, planar gradients of the MEG field distribution were calculated (Bastiaansen & Knosche, 2000). We used a nearest neighbor method where the horizontal and vertical components of the estimated planar gradients were derived, thus approximating the signal measured by MEG systems with planar gradiometers. The planar gradients representation facilitates the interpretation of the sensor‐level data, since the largest signal of the planar gradient typically is located above the source (Nolte, 2003).

Time‐frequency representations (TFR) for absolute power from 3 to 100 Hz were obtained using a fast Fourier transformation (FFT) approach with an adaptive sliding time window three cycles long (ΔT = 3/f; e.g., ΔT = 300 ms for 10 Hz), similarly to previous studies (Bonnefond & Jensen, 2012; Solis‐Vivanco, Jensen, & Bonnefond, 2018). A Hanning taper (also ΔT long) was multiplied by the data prior to the FFT. For the planar gradient, the TFR of power were estimated for the horizontal and vertical components and then summed. The power for the individual trials was averaged over conditions and log‐transformed.

### 2.6 Source analysis

A frequency‐domain beamforming approach based on adaptive spatial filtering techniques (Dynamic imaging of coherent sources; DICS) was used to estimate the absolute power at source level in the entire brain (Gross et al., 2001). We obtained cross‐spectral density matrices by applying a multitaper FFT approach (∆T = 300 ms; 1 Slepian taper resulting in 4 Hz smoothing) on data measured from the axial sensors. For each participant, a realistically shaped single‐shell description of the brain was constructed, based on the individual anatomical MRIs and head shapes (Nolte, 2003). The brain volume of each participant was divided into a grid with a 1 cm resolution and normalized with respect to a template MNI brain (International Consortium for Brain Mapping, Montreal Neurological Institute, Canada) using SPM8 (http://www.fil.ion.ucl.ac.uk/spm). The lead field and the cross‐spectral density were used to calculate a spatial filter for each grid point (Gross et al., 2001) and the spatial distribution of power was estimated for each sensory condition (Attend‐visual/Attend‐auditory) and congruency (congruent/incongruent) in each participant. A common filter was used whenever two conditions were compared (based on the cross‐spectral density matrices of the combined conditions). As for the sensor level analyses, the estimated power was averaged over trials and log‐transformed. The power difference between sensory conditions (visual/auditory) and congruency or time periods was calculated and averaged across participants. For the source reconstruction 33 subjects were included as the MRI of 1 subject was missing. All source data were estimated around 15 Hz according to the peak frequency effect observed in sensor level analyses (see Results section). The source estimates were plotted on a standard MNI brain found in SPM8.

In order to explore the oscillatory dynamics within regions of interest (ROI) of the VAN (see Results section), we used a linearly constrained minimum variance (LCMV) scalar beamformer spatial filter algorithm to generate maps of source activity on a 1 cm grid (Van Veen, van Drongelen, Yuchtman, & Suzuki, 1997). The beamformer source reconstruction calculates a set of weights that maps the sensor data to time‐series of single trials at the source locations, allowing to reconstruct the signal at source level. In addition to TFR of power, we explored the functional connectivity across these reconstructed time series by means of TFR of coherence. In accordance to Nolte et al. (2004), we used the imaginary part of the coherence value, since it is less biased by power. All of our analyses were focused on the time period before the onset of stimuli (i.e., the anticipatory period, during which we expected a suppression of the VAN activity due to top‐down attentional orientation compared with baseline) and the time period after (during which we explored VAN modulations due to a congruency effect between sensory modalities). A 500 ms time window from −700 to −200 ms with respect to the onset of the somatosensory cue was used as baseline (Figure 1). This time window was an appropriate baseline measure as the activity in the frequency range of interest was not modulated during this time, that is, it did not exhibit any anticipatory modulation.

### 2.7 Statistical analysis

Since RT showed normal distributions (Kolmogorov–Smirnov Z for both sensory conditions and congruency modalities ≥0.55, *p* ≥ .41), they were analyzed using repeated measures analysis of variance (ANOVA) (RM‐ANOVA) with condition (Attend‐visual and Attend‐auditory) and congruency (congruent and incongruent) as within‐subject factors. For all described RM‐ANOVA, a Greenhouse–Geisser correction was used in case of violation of sphericity assumption and the Bonferroni test was used for post hoc comparisons.

**FIGURE 2:**
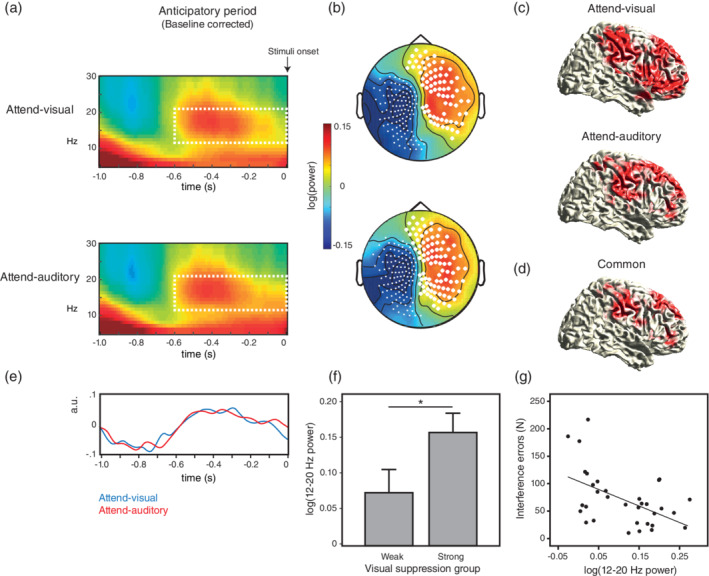
The VAN is suppressed during expectation of relevant stimuli. (a) Time‐frequency representations (TFR) of power during the 1 s before the onset of bimodal (visual/auditory) stimuli for both Attend‐visual and Attend‐auditory conditions, compared with baseline. Data averaged across significant sensors. The dashed rectangles indicate the time‐frequency window (−600 to 0 ms, 12–20 Hz) for subsequent analyses. (b) Results from cluster‐based permutation tests in each condition. Large white dots show the sensors with significant power increases compared with baseline (*p* < .05). Small white dots show significant power reductions. (c) Source representations of the 12–20 Hz increase for Attend‐visual (upper) and Attend‐auditory (lower) conditions. Red areas indicate significant increases compared with baseline after FDR correction (*p* < .01). (d) Common significant areas between both conditions based on a conjunction analysis. (e) Time‐course of 12–20 Hz activity at source level, compared with baseline, averaged across all significant regions for each condition. (f) Participants with less interference errors during the Attend‐auditory condition (strong visual suppressors) showed more 12–20 Hz power at source level (baseline corrected) compared with participants with more errors (weak visual suppressors). The bars represent the averaged values across all significant regions. *p = .05. (g) Higher increase of 12–20 Hz power for both conditions at source level was associated with less total number of interference errors along the task (*p* = .004)

Significant differences of power due to top‐down modulation (i.e., anticipatory vs. baseline time periods in both sensory conditions) or congruency (congruent vs. incongruent) at both sensor and source levels were assessed using a cluster‐based nonparametric randomization test (Maris & Oostenveld, 2007). This test controls for the Type I error rate in situations involving multiple comparisons over sensors, frequencies and times by clustering neighboring sensors, time points and frequency points that show the same effect. For this analysis we included frequencies from 3 to 40 Hz (using 1 Hz increments) with an adaptive time window long enough to include at least 3 cycles in each frequency. We explored from −600 ms to the onset of stimuli for the anticipatory period, and from 200 to 500 ms after for the (post‐stimuli) congruency effect period, based on observed effects at sensor level (see Figures 2a and S2a and S2b. Sensors for which the t value of the difference between conditions exceeded an a priori threshold (*p* < .05) were selected and subsequently clustered based on spatial adjacency, and the sum of the *t* values within a cluster was used as cluster level statistic. The cluster with the maximum sum was used as test statistic. By randomly permuting the data across the two conditions and recalculating the test statistic 2000 times, we obtained a reference distribution to evaluate the statistics significance of a given effect (Monte Carlo estimation). Additionally, for all source level analyses we also conducted a false discovery rate (FDR) correction. This correction allowed us to overcome some limitations of the cluster correction approach such as considering a set of connected smaller cluster (by chance) as one big cluster. Only clusters surviving both the cluster correction and the FDR were reported.

## 3 RESULTS

We used a bimodal (visual/auditory) attentional task that included cueing for relevant stimuli (Figure 1) to quantify the neurophysiological activity associated with active suppression of the VAN activity during top‐down guided attentional allocation. By controlling congruency between the sensory modalities, we also explored the recruitment of the VAN when unexpected relevant (congruent) information arising from the unattended modality was presented, and whether this improved task performance.

### 3.1 Congruent stimuli enhance task performance

Behavioral results were reported before in Solis‐Vivanco, Rodriguez‐Violante, et al. (2018). Briefly, RT analysis showed that subjects were faster for the Attend‐visual compared with the Attend‐auditory trials (834 ± 180 vs. 919 ± 178 ms, respectively, F_[1,33]_ = 83.2, *p* < .001). The RTs also showed a congruency effect, as they were faster for the congruent compared with incongruent trials for both the Attend‐visual and Attend‐auditory conditions (837 ± 171 vs. 947 ± 181 ms, respectively, F_[1,33]_ = 90.3, *p* < .001). Accuracy was better for Attend‐visual compared with Attend‐auditory trials (91 vs. 88%, F_[1,33]_ = 5.63, *p* = .02). Again, a congruency effect was observed, as congruent trials showed better accuracy compared with incongruent (95 vs. 83%, F_[1,33]_ = 76.67, *p* < .001).

In summary, performance was more effective for visual compared with auditory stimuli, as revealed by reduced RT and larger number of correct responses. In addition, congruency between sensory modalities improved performance in both conditions, showing that an involuntary attention enhancement occurred.

### 3.2 VAN activity is suppressed during expectation of relevant stimuli

During the anticipatory period, we observed a power increase in a 12–20 Hz range over right scalp regions before stimuli onset, compared with baseline, in both sensory conditions (Figure 2a). The cluster‐based randomization test controlling for multiple comparisons over time (baseline vs. anticipatory period), frequency (3–40 Hz), and sensors revealed that this difference was significant from 600 ms before stimuli onset in the 12–20 Hz range, regardless of condition (cluster‐level statistic [CS] for Attend‐visual = 3,346, *p* = .014; CS for Attend‐auditory = 4,500, *p* = .011, Figure 2b), and remained significant when combining both conditions (CS = 4,065, *p* = .018).

It is important to note that the cluster test also revealed a significant decrease in the 12–24 Hz range over the left hemisphere (CS for Attend‐visual = −108, *p* = .004; CS for Attend‐auditory = −108, *p* = .001). We interpreted this decrease as reflecting motor anticipation as the mean value of power during the anticipatory period at significant sensors of this cluster was not different between conditions (t_[33]_ = 0.96, *p* = .34), and showed a positive association with RT, regardless of condition and congruency (all *r* ≥ .36, *p* ≤ .04). The increase observed over right sensors could possibly result from a compensatory mechanism of this anticipatory left decrease. However, we performed several analyses that rule out this potential interpretation. First, the two clusters were independent as the power of the left one was not associated with the power of the right one (*r* = .28, *p* = .1, with the trend being in the opposite direction of the prediction) and each cluster showed different time patterns of peak activity (Figure S1a), with the right power cluster reaching its positive peak around −500 ms before the onset of the stimuli, while the left one remained decreasing and reached its lowest value around −260 ms (F_[1,32]_ = 9.72, *p* = .004; Figure S1b). Source localization of the left cluster revealed the participation of premotor, supplementary motor, and parietal areas (Figure S1c), which is in line with the reported beta activity desynchronization observed in these regions during planning of contralateral hand movements (Park, Kim, & Chung, 2013). Finally, since Little, Bonaiuto, Barnes, and Bestmann (2019) reported that pre‐motor beta desynchronization is characterized by the presence of bursts rather than sustained oscillations, we compared the burst rate between both clusters during the anticipatory period (−600 to stimuli onset) according to those authors' method. The right cluster revealed significantly less burst activity compared with the left cluster (t_[33]_ = −4.99, *p* < .001; Figure S1d), implicating that power increases in these regions correspond to stable oscillations, compared with the left cluster. Altogether, this contralateral independent effect might be interpreted in terms of motor preparation, since participants always responded with the right hand (see Tzagarakis, West, and Pellizzer (2015)), rather than an effect of the tactile cue (delivered to one thumb or the other in a counterbalanced way). Therefore, it was not further analyzed. Another potential confounder we investigated was the activity evoked by the somatosensory cue. The time and frequency representation of this activity (measured through the degree of phase alignment across trials, that is, the phase locking factor [PLF]) showed a response in the theta range (3–8 Hz) lasting around 200 ms after its onset and a bilateral distribution exclusively over central sensors corresponding to the somatosensory cortex, with no significant differences between hemispheres (t_[33]_ = 1.33, *p* = .2; see Figure S1E–G). The evoked activity on these sites was not associated with the reported right anticipatory alpha/beta power (*r* = .23, *p* = .20) nor with task accuracy (*r* = −.23, *p* = .19), and no differences were found between participants with strong vs. weak visual suppression (t_[33]_ = −0.47, *p* = .64), unlike what was observed for the right alpha/beta cluster (see below).

The right‐lateralized power increase of 12–20 Hz was further explored at source level (15 Hz) under FDR correction (CS for Attend‐visual = 915, *p* = .005; CS for Attend‐auditory = 1,010, *p* = .004), and revealed significance at right middle and superior frontal, temporal, and parietal regions in both conditions (Figure 2c). We identified the significant areas (surviving FDR correction) from a conjunction analysis with both conditions together (Figure 2d) and sorted them in terms of their statistical value (t) from the highest to lowest. By labeling the top significant areas using the AAL atlas by Tzourio‐Mazoyer et al. (2002), the VAN (inferior frontal gyrus (IFG; MNI [45 30 30]) and temporo‐parietal junction (TPJ, MNI [60–52 34])), the DAN (frontal eye fields (FEF; MNI [40–2 50]) and superior parietal lobule (SPL; MNI [40–50 58])), the right middle frontal gyrus (MFG, MNI [10 38 58]), and supramarginal gyrus (MNI [50–28 36]) remained included. In addition, the signal reconstruction after LCMV filter at these significant areas revealed the 12–20 Hz increase in each condition from 600 ms before stimuli onset (Figure 2e).

In order to test whether this effect was related to task performance, we calculated the average of power values across the grid points with the strongest effect (after FDR correction) in each condition and compared participants with low versus high number of interference errors (i.e., responding to the unattended modality instead of the attended one) in that condition (median split). The *t* tests revealed that participants with less visual interference errors (strong visual suppressors) showed higher 12–20 Hz power (baseline corrected) during the anticipatory period compared with participants with more visual interference errors (weak visual suppressors) under the Attend‐auditory condition (t_[32]_ = −2.0, *p* = .05, Figure 2f). This effect was not observed under the Attend‐visual condition when comparing strong versus weak auditory suppressors (t_[32]_ = −0.56, *p* = .58). We did not find a significant effect of the 12–20 Hz increase on RT for any condition. Also, we explored the association between averaged power values of both conditions and the number of total interference errors along the task. We found that stronger power (baseline corrected) during the anticipatory period was inversely correlated with performance (*r* = −.49, *p* = .004; Figure 2g).

**FIGURE 3:**
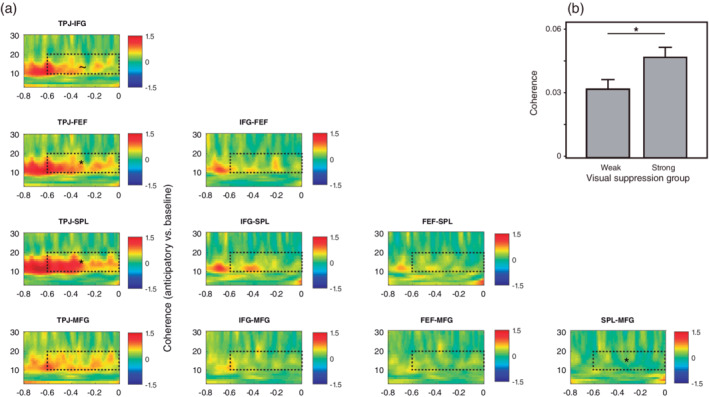
The VAN and DAN networks show increased functional connectivity during top‐down attentional orienting. (a) The TPJ showed increased coherence with FEF and SPL during the anticipatory period in both conditions (displayed averaged and baseline corrected), while SPL and MFG showed a reduction. The dashed rectangles indicate the time‐frequency window of interest. (b) Participants with less interference errors during the Attend‐auditory condition (strong visual suppressors) showed more coherence between significant nodes (TPJ, FEF, and SPL) compared with participants with more errors (weak visual suppressors). The bars represent the averaged values across all significant pairs. ~*p* < .1; *p < .05

When exploring the functional connectivity across relevant ROI (VAN: IFG and TPJ; DAN: FEF and SPL, selection based on power analyses at source level, see Figure 2c) during the anticipatory period, we observed an increase of coherence in the 12–20 Hz range starting from 800 ms before the onset of stimuli with respect to baseline (Figure 3a). Based on the power analyses, we selected a time‐frequency window from −600 ms to stimuli onset in this frequency range and conducted a RM‐ANOVA with the averaged values across time and frequency in each ROI and condition (factor: Attend‐visual vs. Attend auditory), including the baseline period as a comparison reference (factor: baseline vs. anticipatory period). For this analysis, we included the MFG as ROI, given its significant power increase during this time window and the proposed role of this region for connecting the DAN and the VAN (Corbetta et al., 2008). The mean 12–20 Hz difference of power between the anticipatory and baseline periods across conditions and ROI was included as a covariate in this analysis, even when it did not show a significant main effect nor an interaction with any factor. While we did not find differences between conditions (F_[1,31]_ = 0.56, *p* = .46), a significant interaction of ROI by time window (anticipatory vs. baseline, F_[9,279]_ = 6.48, *p* < .0001) revealed significant increases of coherence during the anticipatory period, compared with baseline, of the TPJ with the SPL and FEF (Mean difference [MD] = 0.014, *p* = .004 and MD = 0.008, *p* = .02, respectively; Figure 3a) and also a trend between TPJ and IFG (MD = 0.005, *p* = .09). The SPL and MFG showed a reduction of coherence during this period (MD = −0.009, *p* = .045). Interestingly, participants with less visual interference errors during the Attend‐auditory condition (strong visual suppressors) showed higher coherence between TPJ, SPL, and FEF compared with weak visual suppressors (F_[1,31]_ = 4.47, *p* = .04; Figure 3b). In order to explore the causal relationship between DAN and VAN nodes, we calculated TFR of the phase‐slope index between them for both conditions. Nevertheless, these analyses did not provide reliable results, probably due to small signal‐to‐noise ratios. No further causal analyses were carried out.

In summary, a power increase of 12–20 Hz was observed in right cortical regions before the onset of relevant stimuli, including areas from the VAN and DAN. In addition, higher increase of power in these regions predicted better task performance. Moreover, we observed increased functional connectivity between VAN (especially TPJ) and DAN nodes (SPL and FEF), during this period. Both increases in power and connectivity were stronger in those participants with better ability to filter out distracting visual information.

### 3.3 Alpha power decrease in visual cortex in anticipation of stimuli

Alpha/Beta modulation has been reported more often in sensory networks than in other networks. As reported in Solis‐Vivanco, Rodriguez‐Violante, et al. (2018), we found a power reduction in visual regions starting around 400 ms before the onset of the stimuli, in the 8–15 Hz range (data not shown). This alpha power decrease over visual cortex in anticipation of a visual stimulus was significant compared with baseline (t_[33]_ = −2.52, *p* = .017), and was lower for the Attend‐visual compared with the Attend‐auditory condition (t_[33]_ = −2.53, *p* = .017). Since this result has been extensively described in this previous paper, it was not further analyzed here.

### 3.4 The VAN is recruited after detection of congruency across sensory modalities

**FIGURE 4:**
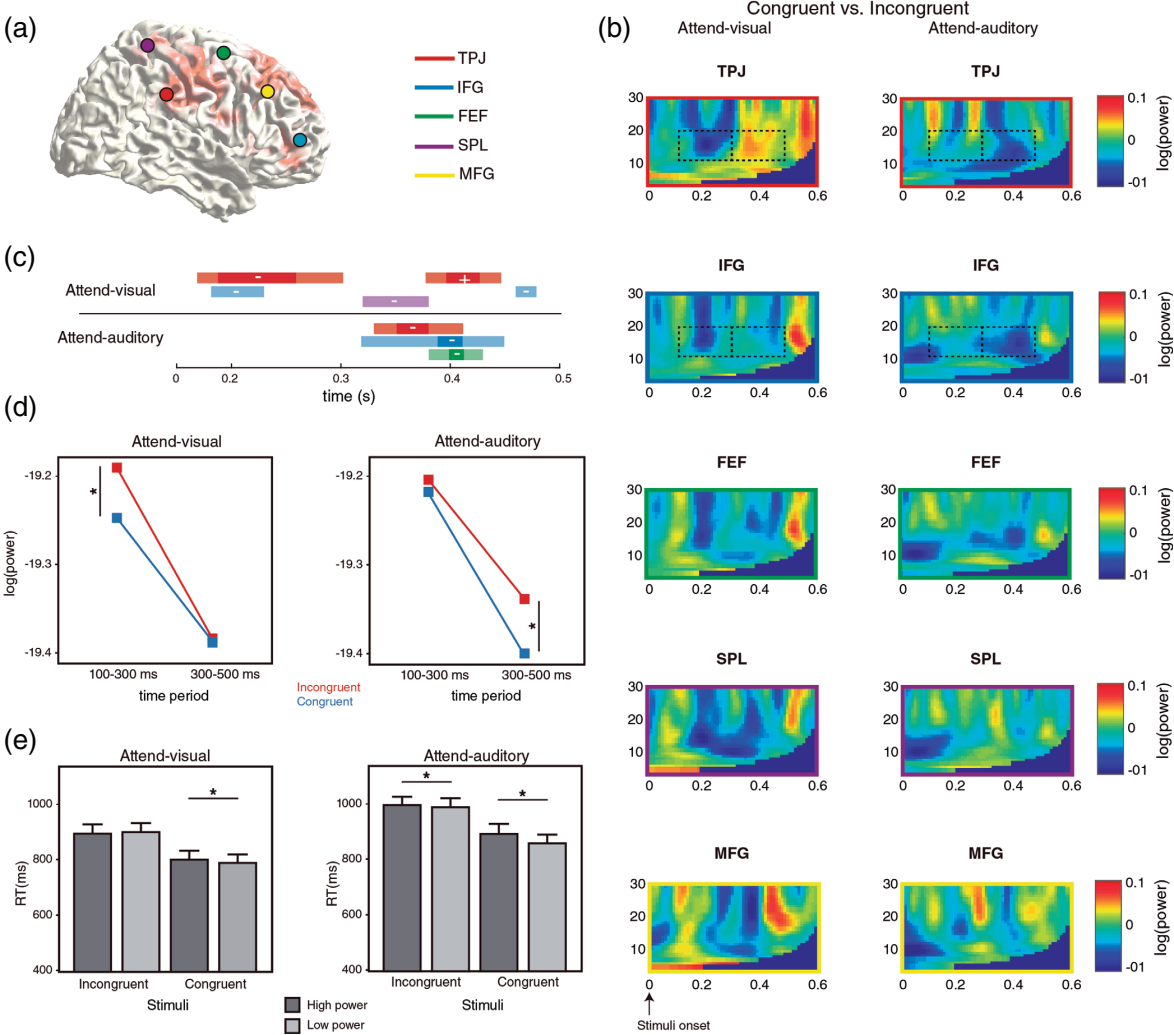
The VAN is recruited after detection of previously unattended congruent stimuli. (a) Specific ROI were selected to explore congruency effects based on the peak differences during the anticipatory period. (b) TFR of congruency effects (congruent vs. incongruent trials) in each ROI including right temporo‐parietal junction (TPJ), inferior frontal gyrus (IFG), frontal eye field (FEF), superior parietal lobule (SPL), and middle frontal gyrus (MFG). An earlier 12–20 Hz power decrease was observed for Attend‐visual vs. Attend‐auditory conditions (dashed rectangles). (c) TPJ and IFG showed a significant congruency effect in both conditions during a 0–500 ms time interval after stimuli onset. Light sections of the bars indicate significant effects (decrease (−) or increase (+)) with *p* < .05. Full‐color sections of the bars indicate *p* < .01 (corrected for multiple comparisons in space). (d) Congruency effects in TPJ and IFG (averaged) were earlier for Attend‐visual compared with Attend‐auditory conditions, in accordance to time‐frequency windows identified in TFR (B). (e) In both conditions, congruent trials with lower 12–20 Hz power at regions showing congruency effects (TPJ and IFG) were associated with reduced reaction time (RT). *p < .05

We explored whether the regions that showed 12–20 Hz power increase during the anticipatory period (VAN and DAN nodes) were also modulated by enhanced attention, that is, elicited by congruency between attended and unattended stimuli. We selected grid points with maximal power differences between the anticipatory period and baseline including both conditions together (although this grid points were also significant for each condition separately) and reconstructed the signal at those points during the congruency period (from stimuli onset to 600 ms afterwards) by means of an LCMV filter. These grid points included the right TPJ, IFG, FEF, SPL, and MFG (Figure 4a). TFRs of these ROIs revealed a clear decrease for congruent compared with incongruent trials in the 12–20 Hz band starting around 150 ms after stimuli onset (Figure 4b). Interestingly, this congruency effect was earlier for Attend‐visual than for Attend‐auditory trials. When conducting *t* tests (corrected for multiple time and space points comparisons) between congruent/incongruent trials along the 0–500 ms time window for each condition and ROI, we found significant effects in TPJ, IFG, FEF, and SPL. The TPJ and IFG were the regions that showed a congruency effect in both conditions (Figure 4c). The latency difference was further explored by comparing averaged power values of VAN ROI (TPJ and IFG) with a RM‐ANOVA that included condition (Attend‐visual/Attend‐auditory), congruency (Congruent/Incongruent), and time window (100–300 and 300–500 ms). This analysis revealed that the congruency effect was earlier for the Attend‐visual condition compared with Attend‐auditory (condition by congruency by time window interaction: F_[1,32]_ = 4.64, *p* = .04; Figure 4d).

Although the congruency effect was not observed at sensory regions under FDR correction, we further explored whether the 12–20 Hz activity was modulated by condition and congruency at visual cortex. After source localizing the signal, we found a congruency effect for the Attend‐visual condition, but not for the Attend‐auditory condition (Condition by congruency interaction: F_[1,32]_ = 3.36, *p* = .07; Attend‐visual: MD = −0.05, *p* = .03; Attend‐auditory: MD = −0.006, *p* = .75; see Figure S3).

We further explored whether the 12–20 Hz power decrease was associated with task performance (Figure 4e). Our hypothesis was that trials with low alpha/beta power (i.e., higher activation of the VAN) would allow an enhanced congruency effect and consequently faster performances, compared with trials with high power (lower VAN activation). To this end, we classified the trials in each participant as showing low or high power in each condition and congruency variant (based on a median split) from the grid points that showed a congruency effect at source level (average of TPJ and IFG). Then we compared the RT among power, condition, and congruency factors, although ignoring the congruency main effect, already known as significant. A RM‐ANOVA revealed that congruent trials with low power showed shorter RTs compared with those with high power (Power by congruency effect: F_[1,32]_ = 6.86, *p* = .013; MD for congruent trials = −24.0, *p* = .01; MD for incongruent = −0.42, *p* = .96). Also, an interaction of condition by power was found (F_[1,32]_ = 7.03, *p* = .01). Post hoc comparisons revealed that trials in general with low power reduced RT within the Attend‐auditory condition (MD for Attend‐auditory = −22.1, *p* = .01; MD for Attend‐visual = −2.34, *p* = .74; Figure 4e).

When assessing the power congruency effect both at sensor and source levels for the whole brain, the 12–20 Hz decrease was further confirmed at similar right sensors as for the anticipatory period (Figure S2a). The cluster‐based randomization test in a 200–500 ms time window showed that this effect was especially prominent for the Attend‐auditory condition at sensor level (Attend‐visual: CS = −16, *p* = .05; Attend‐auditory: CS = −41, *p* = .013; Figure S2b). When exploring this effect at source level (LCMV filter and power averaged values at 15 Hz), both conditions revealed this effect on right superior and inferior frontal, temporal and parietal areas (300–400 ms; Attend‐visual: CS = −346, *p* = .037; Attend‐auditory: CS = −316, *p* = .048) and with a similar pattern when considering both conditions together (Figure 2c). After an FDR correction, nodes from the VAN (TPJ and IFG) remained included. At both sensor and source levels, the topographic profiles of the right sided 12–20 Hz modulation during the anticipatory (compared with baseline) and post‐stimuli (congruent vs. incongruent) periods were notably similar (Figure 2d). In order to discard that the congruency effect rather reflected differences related to the overrepresentation of incongruent vs. congruent stimuli (75 vs. 25%), we explored whether congruent trials evoked the P300 event‐related field (P300m) at parietal regions due to an oddball effect (Polich, 2007). Nevertheless, this ERF was not observed for any condition (data not shown).

**FIGURE 5:**
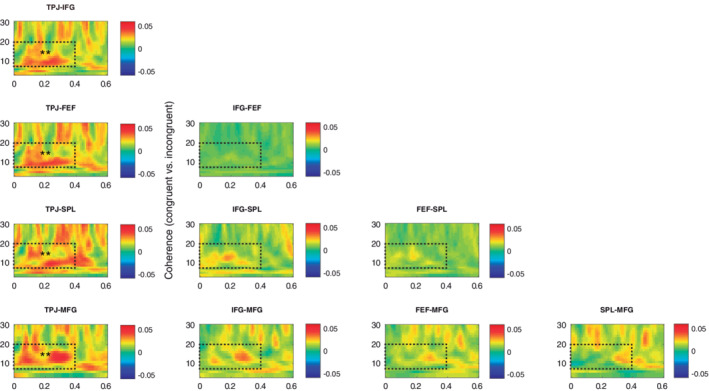
The VAN and DAN show increased functional connectivity after detection of previously unattended congruent stimuli. The TPJ showed increased coherence with FEF, SPL, and MFG after congruent stimuli onset compared with incongruent ones in both conditions (displayed averaged and baseline corrected). The SPL also showed increased coherence with IFG and FEF. The dashed rectangles indicate the time‐frequency window of interest. *p < .05, **p ≤ .01

When exploring with a RM‐ANOVA the functional connectivity across relevant ROI (TPJ, IFG, FEF, SPL, and MFG) during the congruency period, we found a significant increase of coherence for congruent vs. incongruent stimuli in the alpha‐beta range (8–20 Hz, F_[1,31]_ = 12.99, *p* = .001; Figure 5), regardless of condition. A significant interaction of congruency by pair (F_[9,279]_ = 3.29, *p* = .013) revealed that this difference was particularly significant between TPJ and the rest of the ROIs (IFG: MD = 0.01, *p* = .002; FEF: MD = 0.01, *p* = .001; SPL: MD = 0.01, *p* = .007; MFG: MD = 0.012, *p* = .008), between IFG and SPL (MD = 0.006, *p* = .03), and between FEF and SPL (MD = 0.005, *p* = .028). The mean 8–20 Hz difference of power between congruent and incongruent trials across conditions and ROI was included as a covariate in this analysis, even when it did not show a significant main effect or an interaction with the congruency factor. No associations were found between coherence in these areas and task performance.

In accordance to a recent report showing an increase of gamma oscillations (>40 Hz) within the VAN after the involuntary detection of auditory distracting stimuli (ElShafei et al., 2018), we explored whether there was a congruency effect within the 50–80 Hz range after stimuli onset in the previously selected ROI. Nevertheless, no congruency effect was observed at this frequency range (Figure S3).

In summary, we found a power decrease, earlier in the visual condition, in the same frequency range as the anticipatory period in the VAN (TPJ and IFG), but also in the DAN (FEF and SPL), for congruent compared with incongruent trials. Such decrease predicted performance speed. In addition, increased connectivity between VAN and DAN nodes was observed for congruent trials. These results suggest an involvement of the two networks reflecting effective attentional enhancement after unexpected detection of relevant information in previously unattended sensory modalities.

## 4 DISCUSSION

In the present study, we aimed to determine (a) the oscillatory profile of the suppression of the VAN during top‐down oriented attention processes and (b) whether this network was recruited when the information presented in an unattended sensory modality was congruent with the information presented in the attended modality, a special case of attention enhancement, in order to improve task performance. We found a power increase of alpha/beta (12–20 Hz) oscillations in the VAN during top‐down attentional orientation. This increase was associated with better task performance, suggesting that the VAN suppression might prevent shifting attention to distractors. Moreover, the TPJ was synchronized with the FEF in the same frequency band, suggesting that the dorsal attention network (DAN) might participate in the suppression of the VAN. In addition, we found a 12–20 Hz power decrease and enhanced synchronization, in both the VAN and DAN, when information from one sensory modality was congruent with the other, suggesting an involvement of both networks when attention is involuntarily enhanced due to multisensory integration.

First, we observed an increase of oscillations in the 12–20 Hz frequency band in VAN and DAN nodes following the presentation of the cue indicating the modality to attend, that is, during top‐down attention. Such increase was correlated with better behavioral performances over participants, as indexed by a decrease in the number of interference errors in incongruent trials (although this correlation should be taken cautiously due to the number of participants Schönbrodt & Perugini, 2013). These results suggest that the VAN is suppressed during top‐down attention processes, as showed by fMRI papers that investigated this question (Shulman et al., 2003; Shulman et al., 2007; Todd et al., 2005) and is in line with the hypothesis that this suppression would reflect a mechanism allowing to protect goal‐driven behavior from distractors. We show here that such suppression is expressed in a broad frequency band related to high alpha/low beta oscillations, that is, in a higher frequency band compared with the frequencies observed in sensory networks. Importantly, the same frequency range was modulated during sensory processing (see below). However, modulations in low beta over sensory regions have also been reported during attentional tasks (e.g., van Ede, Koster, and Maris (2012) and Siegel et al. (2008)). Moreover, a modulation in a similar frequency range (10–20 Hz) in and between nodes of the VAN and DAN has been reported during a visual search task (Spaak, Fonken, Jensen, & de Lange, 2016) as well as in anticipation of or during the processing of matching stimuli in multisensory paradigms (Goschl, Friese, Daume, Konig, & Engel, 2015; Misselhorn, Friese, & Engel, 2019; Wang, Goschl, Friese, Konig, & Engel, 2019). Interestingly, a comprehensive study has demonstrated that alpha‐beta frequency peaks differ across regions and experimental designs (Haegens, Cousijn, Wallis, Harrison, & Nobre, 2014) (see also ElShafei et al. (2018)). Altogether, these results indicate that the frequency range of alpha (and beta) oscillations might diverge between brain regions or between tasks although they might still be associated with a similar mechanism, for example, functional inhibition. Further studies are required to understand whether these differences result for example, from the anatomical connectivity of the networks involved and/or from the requirement for multi‐timescale processing according to the cognitive process involved.

Furthermore, we observed a stronger coherence between the nodes of the VAN network as well as between FEF (part of the DAN) and TPJ during the anticipatory period. Interestingly, participants with stronger ability to suppress visual distractors showed higher connectivity across these nodes. This latest result could provide evidence in favor of the idea that the suppression of the VAN is driven by the DAN (Shulman et al., 2003). Nevertheless, since we did not find a clear direction of such connectivity between VAN and DAN (possibly due to a reduced signal‐to‐noise ratio), this hypothesis should be taken with caution. On the other hand, we found a decrease in connectivity between MFG and DAN (SPL) during top‐down attention. This was unexpected, since the right posterior MFG has been discussed as another candidate region for linking the dorsal with the ventral system (Corbetta et al., 2008). Further research is needed to confirm and understand our result.

At first sight, it could be considered surprising that we did not report an increase of alpha oscillations in sensory regions in anticipation of distractors (see Solis‐Vivanco, Rodriguez‐Violante, et al. (2018)), while we do report such an increase (in a higher frequency range) in VAN and DAN networks. These are, however, not conflictual results. While we discuss above why alpha oscillations increase in VAN/DAN would be expected in our task, there are three main reasons potentially explaining the absence of an alpha increase in sensory regions in the current task. First, as discussed in Solis‐Vivanco, Rodriguez‐Violante, et al. (2018), our task is not designed for showing an anticipatory alpha increase since in 25% of the trials the auditory and visual stimuli were congruent, leading to attentional enhancement, and dampening of the distracting value of the unattended stimulus. Second, while sensory alpha oscillations have been related to inhibitory processes in sensory areas in for example, working memory tasks (Bonnefond & Jensen, 2012; Rosner, Arnau, Skiba, Wascher, & Schneider, 2020; Schneider, Goddertz, Haase, Hickey, & Wascher, 2019), there is currently a hot debate regarding the presence of such a sensory alpha increase during attentional tasks (e.g., Antonov, Chakravarthi, & Andersen, 2020; Foster & Awh, 2019; Zhigalov & Jensen, 2020). The alpha power decrease in anticipation of relevant stimuli appears more robust in the literature although this effect relies on the likelihood or the timing of the target appearance (Capilla, Schoffelen, Paterson, Thut, & Gross, 2014; Gould, Rushworth, & Nobre, 2011; Ikkai, Dandekar, & Curtis, 2016; Kelly, Gomez‐Ramirez, & Foxe, 2009; Sauseng et al., 2005; Yamagishi, Goda, Callan, Anderson, & Kawato, 2005). In line with the literature, we did observe an anticipatory alpha decrease in visual regions (not in auditory cortex though, but see Solis‐Vivanco, Rodriguez‐Violante, et al. (2018), for a discussion, and Mazaheri et al. (2014)) both compared with baseline and during visual attention compared with auditory trials. Third, the absence of alpha increase in visual regions compared with baseline might result from a strong visual alpha power during baseline, given that the cue was in the somatosensory domain and strong visual alpha might be necessary to optimally process the cue (see Haegens, Nacher, Luna, Romo, and Jensen (2011)).

We further observed that the VAN was recruited, as indexed as well by a power decrease in the 12–20 Hz band, when a congruent stimulus was presented in the unattended sensory domain. Importantly, during stimuli processing and when comparing congruent versus incongruent trials there was no evidence of power modulation on left regions as observed during the anticipatory period, which supports the attentional role of this network, rather than a motor one. The congruency effect is a special case of attentional enhancement, as relevant information is still present in the attended dimension though its saliency is increased due to multisensory integration, and includes a consequent behavioral benefit (Gau et al., 2020; Van der Burg, Olivers, Bronkhorst, & Theeuwes, 2008). Importantly, this effect remains even when congruent trials are rare (Van der Burg et al., 2008), resembling oddball paradigms under which the VAN is usually activated (Kim, 2014). Furthermore, the VAN alpha/beta decrease predicted response speed in both conditions and was observed earlier in the Attend‐visual condition, that is, when the unattended stimulus was presented in the auditory domain, compared with the Attend‐auditory condition. The processing of auditory stimuli has been shown to be faster than processing of visual ones, which could explain the earlier activation of the VAN in the Attend‐visual condition (Pain & Hibbs, 2007). However, it should be noted that reaction times were faster in the Attend‐visual condition, congruency effects were stronger for the Attend‐auditory condition at sensor level, and VAN suppression was better in the strong visual suppressors during the anticipatory period. These results may reflect the sensory dominance of the visual domain, and hence the need for more effective modulation (suppression/recruitment) of the VAN for this type of information.

Interestingly, the DAN was also more activated during congruent than incongruent trials after an early decrease in IFG and TPJ, although only FEF reached the significant level after multiple comparison corrections in the Attend‐auditory condition. In line with this, previous fMRI work has also reported higher FEF activity during reorienting of attention (e.g., Corbetta & Shulman, 2011; Vossel, Thiel, & Fink, 2006). Although the timing of activation of the different networks would need to be further investigated, it seems that the VAN was further activated by congruency earlier than the DAN, which ultimately might guide attention toward the unattended sensory domain in addition to the attended domain. In line with this, Proskovec et al. (2018), reported a late synchrony in the alpha band in the DAN, possibly associated with reorienting of attention, although they also reported an early activation of FEF. Interestingly, in the Attend‐visual condition, we observed an increase of the oscillations in the 12–20 Hz band in the TPJ following the decrease, possibly reflecting a suppression of this node after its recruitment. The time window we could analyze did not allow to determine whether a similar increase was later observed in the Attend‐auditory condition.

We also found a congruency effect in the visual cortex for the Attend‐visual condition, although it did not survive multiple comparisons corrections. The congruency effect only in the Attend‐visual condition, not in the Attend‐auditory condition, over the visual cortex (i.e., the relevant area) suggests that congruency further enhances the processing of the attended stimulus and not necessarily of the unattended stimulus. Interestingly, such enhancement operates in the same frequency range as the one in VAN and DAN nodes, possibly facilitating communication between sensory and attentional networks. Further investigation will be required to test this hypothesis.

In addition, we observed a coherence increase for congruent trials within and between VAN and DAN (TPJ, IFG, FEF, and SPL). This alpha/beta synchrony could reveal the mechanism allowing the interaction within and between these networks during involuntary attentional enhancement (Vossel et al., 2014), though we did not find a direct association with task performance. It should be noted that our selection of ROI for congruency comparisons was based on significant areas detected during the anticipatory period. Thus, other relevant regions involved in task performance and potentially synchronized with VAN and DAN during congruency detection may not have been considered. Future studies might explore connectivity patterns time‐locked to neural responses after involuntary attention increase in order to elucidate this possibility.

We did not find any significant difference between congruent and incongruent trials in other frequency bands, neither in the theta band as reported by Proskovec et al. (2018) nor in the gamma band as reported by ElShafei et al. (2018). These discrepancies could be related to the paradigms across studies. Proskovec et al. used a Posner cueing task in which invalid trials (target on the uncued side) appeared 50% of the trials and could be very quickly detected. Possibly the theta increase they observed was more related to a bottom‐up process, locked to the stimulus onset (see e.g., van Kerkoerle et al. (2014) showing that theta oscillations might be related to feedforward activity). In our study, the mechanism of attentional enhancement might be more complex as it requires the detection of congruency (i.e., incorporating relevance from the unattended domain while holding it in the attended one). Although we observed a clear gamma increase in all the nodes (VAN and DAN) compared with baseline, we did not observe a gamma power difference between congruent and incongruent trials. Again, a difference in terms of paradigm might explain the discrepancy with ElShafei et al. study. In their paradigm, the auditory stimuli inducing a change in the VAN were not relevant to the task, unlike to the present study. It might be therefore interesting to study the role of relevance versus saliency for VAN activation in the different frequency bands. For instance, it has been reported that irrelevant, novel auditory stimuli generate a reduction of power in the alpha/beta band at parietal regions (Solis‐Vivanco, Rodriguez‐Violante, et al., 2018). Nevertheless, whether the source of this decrease includes the VAN (and DAN) remains to be explored. In addition, future studies might explore the role of disengagement (i.e., full switch from attended to unattended domain) over DAN and VAN activation, which was explored in the Posner task used by Proskovec et al. (2018), but not necessarily present in our study.

As a final remark, we hypothesized that the decrease of the BOLD signal observed in the VAN network during similar tasks in fMRI (Shulman et al., 2003; Shulman et al., 2007; Todd et al., 2005) would be reflected in an increase of alpha/beta oscillations as observed over sensory areas (Haegens et al., 2011; Sadaghiani et al., 2012). We therefore considered the observed increase of alpha/beta power in the VAN as potential evidence of inhibition of the activity of this network. While the top‐down role of alpha/beta activity, mainly in the DAN and sensory hierarchy, has been reported in the literature (Bastos et al., 2015; Michalareas et al., 2016), and without necessarily alluding a potential inhibitory role (Lobier, Palva, & Palva, 2018), we suggest that both possibilities (i.e., top‐down regulation and functional inhibition) are not mutually exclusive. This is particularly true when we consider the activity of the VAN in addition to, or more specifically in interaction with, the activity of the DAN. We propose that changes in alpha/beta power and synchronization indicates the involvement of the VAN in both reducing interference from distractions (alpha/beta power increase during delay) and extracting relevant information from unattended channels (alpha/beta decrease in the congruent condition), in both cases in interaction with the DAN. We therefore consider the changes in alpha/beta power in the VAN as well as the increased connectivity with the DAN as correlates of attentional top‐down guidance through functional inhibition.

Among the limitations of our study, we did not explore the VAN and DAN effects at sensory regions. A recent fMRI study by Rossi, Huang, Furtak, Belliveau, and Ahveninen (2014) showed increased connectivity between auditory cortex and different nodes of the DAN and VAN during cued voluntary and novelty‐driven auditory orienting, respectively. In addition, our sample included only young adults. Future research might explore the VAN and DAN modulation during attentional orientation and involuntary enhancement along development, including children and older adults. In addition, how these networks can be compromised in patients with neurologic and psychiatric disorders with attention impairment remains to be explored.

In conclusion, our results show that effective attentional allocation, regardless of sensory modality, includes the modulation and cooperation between ventral and dorsal attention networks through upper‐alpha/beta oscillations.

## CONFLICT OF INTERESTS

The authors declare no competing financial interests.

## ETHICS STATEMENT

This study fulfilled the Declaration of Helsinki criteria and was approved by the local board ethics committee at the Donders Institute for Brain, Cognition, and Behaviour, Radboud University.

## Supporting information


**Appendix** **S1**: Supporting Information

## Data Availability

All data are available by request to the authors.
