# Supplementary material for: New insights on the ventral attention network: Active suppression and involuntary recruitment during a bimodal task
Source: Hum Brain Mapp. 2020 Dec 21;42(6):1699–713. doi: 10.1002/hbm.25322 (PMC7978122; doi:10.1002/hbm.25322)
Supplement: Supplementary file 1 — Appendix S1: Supporting Information [file HBM-42-1699-s001.docx]

**SUPPLEMENTARY FIGURES**


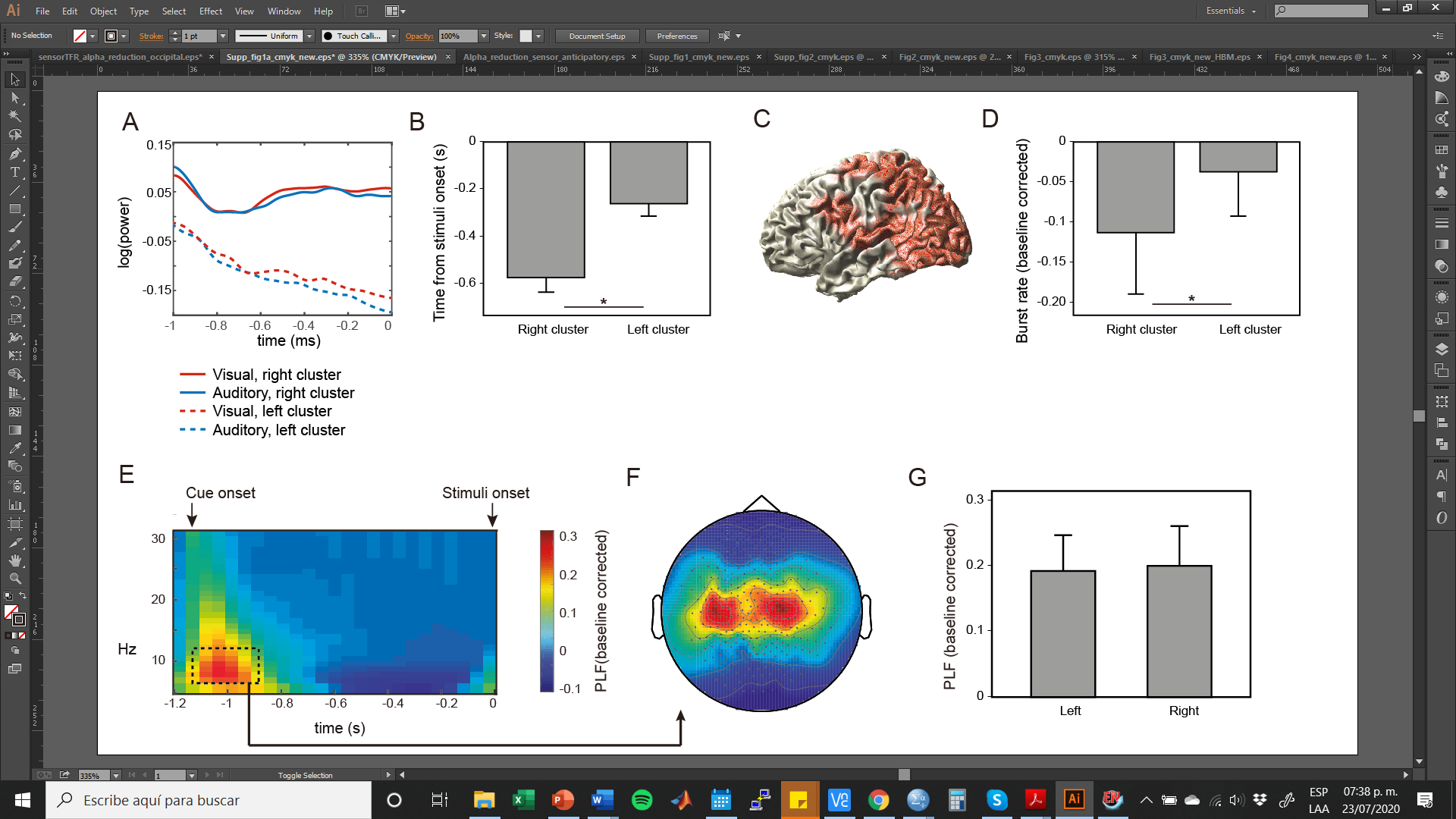


Supplementary figure 1. (A) Time course of the left and right clusters observed during the anticipatory period, showing different temporal patterns. (B) Peak activity before the onset of targets was significantly different between left and right clusters. *p<0.01. (C) Source localization of the left cluster showed clear involvement of premotor, supplementary motor, and parietal areas, which were not included in the right cluster. Red areas indicate significant increases compared to baseline after FDR correction (p<0.01). (D) Burst analysis (as reported by Little et al. (2019) for pre-motor beta desynchronization) showed reduced burst rate during the anticipatory period in the right cluster compared to the left one. *p<0.01. (E) The time-frequency representation of the activity evoked by the cue (measured through the degree of phase alignment across trials, i.e. the phase locking factor (PLF)) shows a response in the theta range (3-8 Hz) lasting around 200 ms after its onset and (F) a bilateral distribution exclusively over central sensors corresponding to the somatosensory cortex, with no significant differences between hemispheres (t=1.33, p=0.2; G).


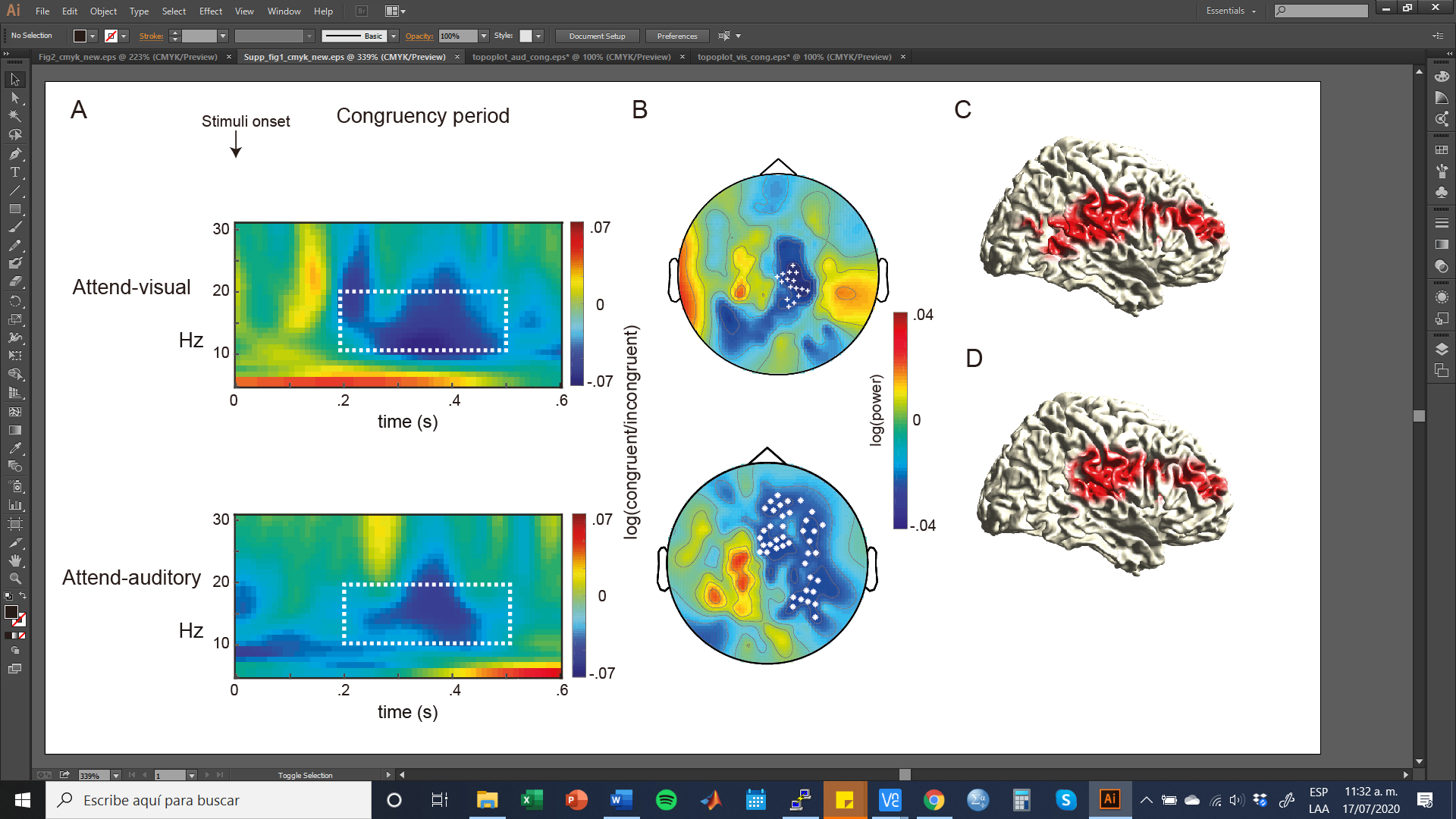


Supplementary figure 2. Detection of congruent information in unattended sensory modalities reduces upper alpha/low beta oscillations. (A) A 12-20 Hz decrease around 200 ms was observed after stimuli onset during congruent trials compared to incongruent trials. Time-frequency plots represent the average of sensor showing significant effects. (B) Significant decreases for both conditions were observed at right frontal, temporal, and parietal sensors. +p=0.05, *p<0.05. (C) After an FDR correction at source level, nodes from the VAN (TPJ and IFG) were observed to show power decreases for congruent trials when considering both conditions together. Red areas indicate significant decreases compared to incongruent trials (p<0.05). (D) The topographic profiles of the right sided 12-20 Hz increase during the anticipatory (compared to baseline) and decrease during the post-stimuli (congruent vs. incongruent) periods were notably similar. Red areas indicate common significant regions between both time periods.


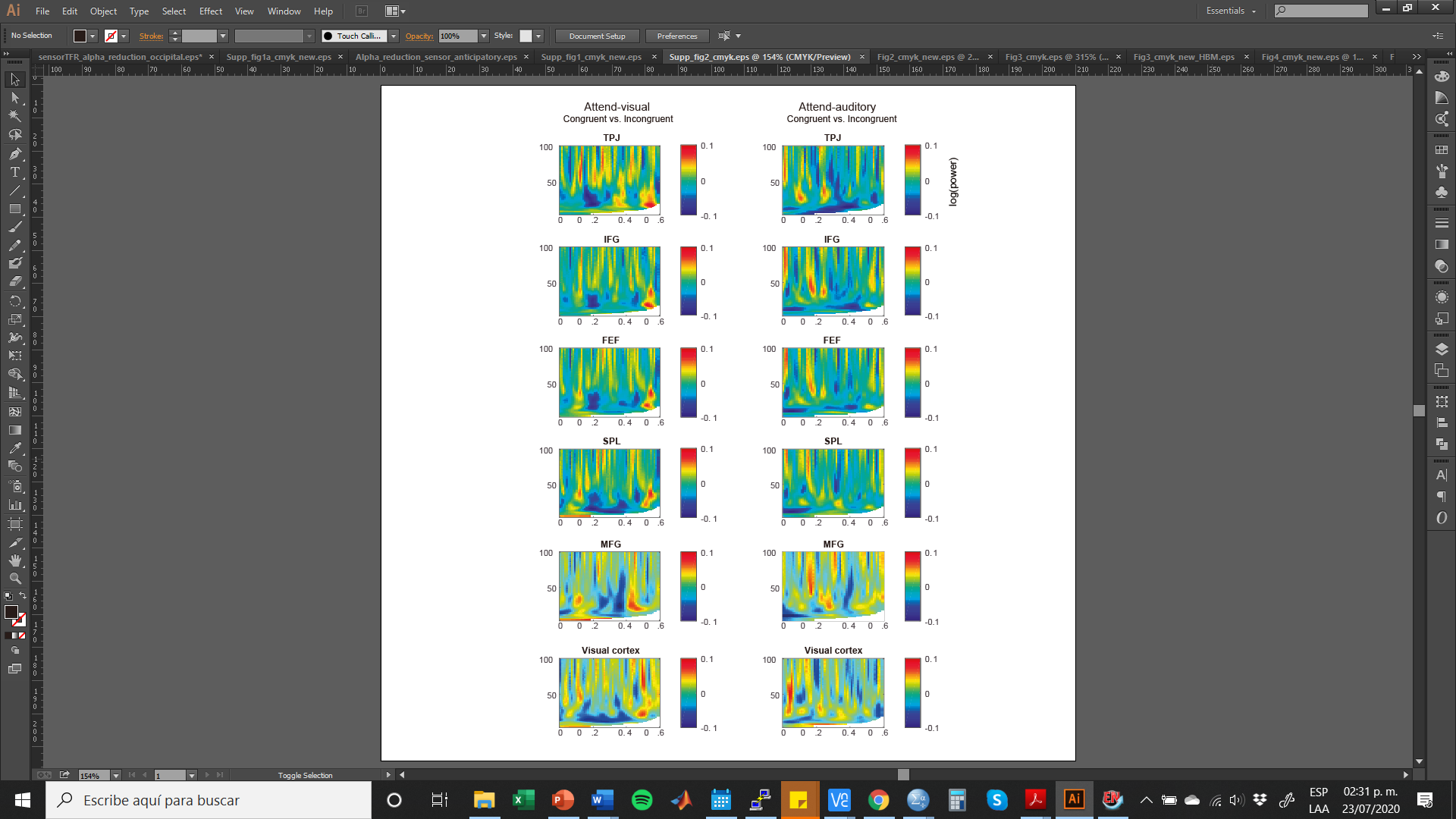


Supplementary figure 3. Congruency effects (congruent vs. incongruent) were not observed in the gamma range (40-80 Hz) at any ROI or condition. An additional analysis on visual cortex revealed a congruency effect in the 12-20 Hz range, exclusively for the Attend-visual condition (p=0.03).
